# Involvement of Phytochrome-Interacting Factors in High-Irradiance Adaptation

**DOI:** 10.3390/ijms262311660

**Published:** 2025-12-02

**Authors:** Pavel Pashkovskiy, Anna Abramova, Alexandra Khudyakova, Mikhail Vereshchagin, Vladimir Kuznetsov, Vladimir D. Kreslavski

**Affiliations:** 1 K.A. Timiryazev Institute of Plant Physiology, Russian Academy of Sciences, Moscow 127276, Russia; pashkovskiy.pavel@gmail.com (P.P.);; 2 Institute of Basic Biological Problems, Russian Academy of Sciences, Pushchino 142290, Russia

**Keywords:** high-intensity light, *Arabidopsis thaliana*, gene expression, photosynthesis, chlorophyll fluorescence, oxidative stress

## Abstract

Phytochrome-interacting factors (PIFs) are key transcriptional regulators of phytochrome signalling that coordinate photomorphogenesis and photosynthesis under different environmental conditions. PIFs play an important role in this regulation and act mainly as negative regulators of photomorphogenesis, but under high-intensity light (HIL), their functions can also include adaptive roles. We investigated the contribution of individual PIFs to the adaptation of the photosynthetic apparatus in wild-type *A. thaliana* and *pif4, pif5*, *pif4pif5,* and *pif1pif3pif4pif5* mutants exposed to HIL for 0, 16, 32, or 48 h. Chlorophyll fluorescence parameters (Y(II), F_v_/F_m_, NPQ), net photosynthesis (Pn), transpiration rates, stomatal conductance (g_S_), pigment contents and the expression of key genes were evaluated. The response of plants to HIL varied depending on the duration of exposure. After 16 h of irradiation, the greatest reductions in Pn and g_S_ were observed in the *pif4pif5* and *pif1pif3pif4pif5* mutants, whereas after 48 h, the decreases were most pronounced in the *pif4*, *pif5*, and *pif4pif5* mutants. After 16 h of HIL exposure, the absence of *pif4* and *pif5* did not substantially alter the chlorophyll fluorescence parameters. However, after 48 h, both Y(II) and Fv/Fm were lower in these mutants than in the wild type, indicating changes in PSII functional status rather than direct reductions in photochemical quantum efficiency. At 16 h, chlorophyll levels were the highest in *pif5* and WT, whereas anthocyanin and UV-absorbing pigment (UAP) levels were the highest in *pif4*, *pif5* and WT. After 48 h, the highest levels of any pigments were detected in the WT and the *pif1pif3pif4pif5* mutant. These results suggest that the accumulation of anthocyanins and UAPs under HIL is likely associated with the regulation of transcription factors, such as PIFs, de-etiolated 1 (DET1), constitutive photomorphogenic 1 (COP1), and elongated hypocotyl 5 (HY5). During prolonged HIL exposure, the absence of PIF4 and PIF5 has a critical impact on photosynthesis and the accumulation of photosynthetic pigments, whereas the simultaneous loss of PIF1, PIF3, PIF4, and PIF5 is less detrimental. This finding likely indicates opposite roles of PIF1 and PIF3 in the above-described processes, on the one hand, and PIF4 and PIF5, on the other hand, under HIL conditions.

## 1. Introduction

Light quality and intensity are crucial for plant growth and photomorphogenesis, helping plants adapt to changing environmental conditions. Various photoreceptors participate in the perception of light cues, among which red- and far-red-absorbing phytochromes play a central role in regulating developmental and metabolic processes, including de-etiolation, shade avoidance, and adaptation to environmental stresses [1,2]. The phytochrome family comprises two functional types and five members (PHYA–E): the type I phytochrome PHYA is light-labile and predominantly absorbs far-red light, whereas type II phytochromes (PHYB–E) are light stable and remain in their active Pfr conformation under high red/far-red light ratios. Among them, PHYB is the principal receptor of red light and mediates low-fluence responses and shade avoidance [3].

During the course of evolution, complex signalling networks have been formed that ensure the perception of and adaptation to changes in the quality, intensity and duration of lighting [4]. The most important components of these networks are phytochrome-interacting factors (PIFs), a family of basic helix-loop-helix (bHLH) class transcription factors that integrate light signals with physiological and metabolic processes in plants [5].

Most PIFs function as negative regulators of photomorphogenesis [6]. All seven known family members in *A. thaliana* (PIF1, PIF3, PIF4, PIF5, PIF6, PIF7, and PIF8) are capable of binding to the active form of phytochromes. Light-activated phytochromes induce the degradation of PIFs or block their interaction with DNA, which relieves the repression of light-dependent genes that induce photomorphogenesis [7,8,9]. Moreover, individual PIFs perform specialized functions; for example, PIF1 inhibits seed germination by activating ABA-, auxin-, and jasmonate-dependent pathways [9,10], and PIF4 regulates hypocotyl elongation and flowering at elevated temperatures [11]. Under shaded conditions, PHYB inactivation leads to the accumulation of PIF3, PIF4, and PIF5, as well as the activation of PIF7, which initiates flowering induction under competition with neighbouring plants [12,13,14].

The functional diversity of PIFs is determined by their different stabilities, affinities for phytochromes, and interactions with other transcription factors [15]. For example, Elongated Hypocotyl 5 (HY5) and PIF form a dynamic module sensitive to changes in light and temperature [16]. HY5 stimulates the biosynthesis of photosynthetic pigments, whereas PIFs inhibit their biosynthesis. However, HY5 and PIFs do not always function as antagonists. For example, PIF3 and HY5 can act synergistically, activating the transcription of the same anthocyanin biosynthetic genes [17]. In addition, PIFs modulate hormonal pathways, including abscisic acid (ABA), gibberellins (GA), and ethylene-dependent pathways [18,19], and are also involved in the regulation of stress responses such as drought, high temperature and salt stress [20,21]. Thus, PIF4 and phytohormones jointly regulate the development of adaptations to heat, salt and water deficiency [19]. At the molecular level, PIFs control the expression of key genes involved in photomorphogenesis and plant defence systems. It has also been shown that PIF proteins are subject to complex multilayer regulation, including changes in their abundance, phosphorylation state, and spectral behaviour under light of different spectral compositions [22]. Their activity and stability strongly depend on phosphorylation, which controls PIF degradation following phytochrome activation and thus mediates light-dependent transcriptional responses. Furthermore, microRNAs have been suggested to modulate PIF expression and influence downstream signalling pathways, providing an additional layer of posttranscriptional regulation under varying light and stress conditions [22,23].

Chlorophyll fluorescence parameters such as Fv/Fm and Y(II) have long been used as functional indicators of PSII status under different environmental conditions [24]. However, recent studies have shown that these parameters cannot be interpreted as true photochemical yields of PSII and must be used with caution [25,26].

Despite the accumulated data, the role of PIFs in the regulation of photosynthetic activity and tolerance to high-intensity light (HIL) remains poorly understood. In particular, little is known about how individual PIFs perform unique or overlapping functions at different stages of plant adaptation to HIL; in particular, PIFs affect photochemical and metabolic processes in adult plants.

In this study, the mutants *pif4*, *pif5*, *pif4pif5*, and *pif1pif3pif4pif5* were used to comprehensively analyze the contribution of individual family members to the adaptation of *A. thaliana* to the HIL. The initial assumption was that PIFs perform both unique and partially redundant functions, regulating antioxidant and photoprotective processes, as well as photosynthetic activity and pigment accumulation at the early and late stages of the adaptation of *A. thaliana* to HIL.

## 2. Results

### 2.1. Plant Material

Compared with those of the WT and mutants, shortened petioles and increased rosette compactness with increased leaf blade area were observed. In the *pif4* and *pif5* mutants, the leaves were wider, and the rosette density was denser, but the overall morphology was similar to that of the WT and mutants. In the *pif4pif5* mutants, a more compact rosette with an increased leaf blade area was formed. The *pif1pif3pif4pif5* quadruple mutant presented the most pronounced difference: the leaves were virtually devoid of petioles, resulting in an increase in the photosynthetic leaf surface area due to the expansion of the blades. Furthermore, the quadruple mutant leaves were characterized by greater thickness and leaf mechanical strength (Figure S1).

### 2.2. Photosynthetic Activity and Chl Fluorescence Parameters

**Figure 1:**
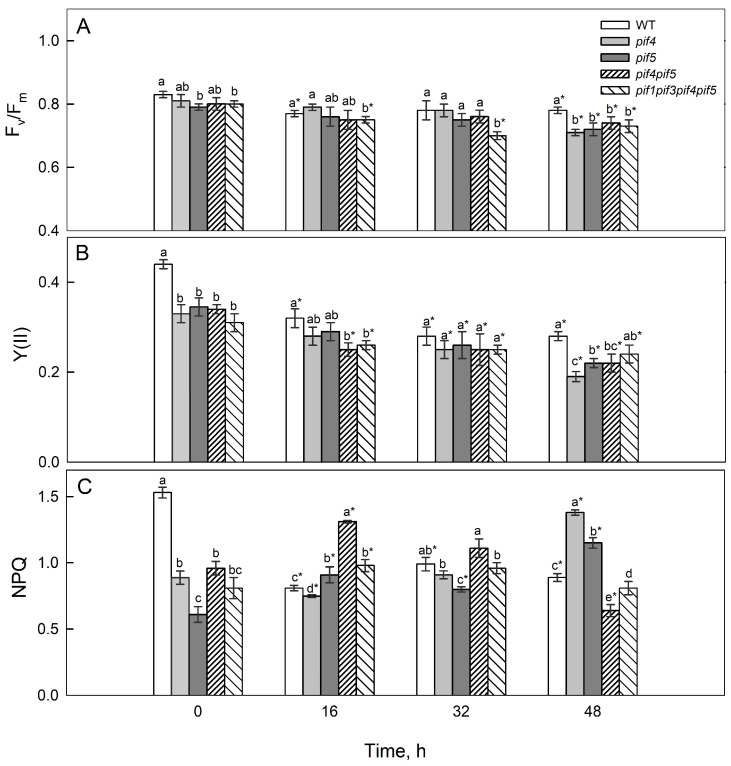
(**A**) Maximum chlorophyll fluorescence parameter Fv/Fm and (**B**) effective chlorophyll fluorescence parameter Y(II), which are commonly used as functional indicators of PSII status, and (**C**) nonphotochemical quenching (NPQ) and in the leaves of *A. thaliana pif* mutants after 16, 32 and 48 h of HIL treatment. Different letters (a–e) indicate significant differences (*p* ≤ 0.05) according to ANOVA on ranks followed by the Duncan method. Asterisks indicate significant differences within the same genotype at 16, 32, and 48 h (*p* < 0.05; Student’s *t* test). The mean values ± SE are shown, *n* = 7.

Initially, before irradiation, the values of the chlorophyll fluorescence parameter Fv/Fm, which is widely used as a functional indicator of PSII status, differed only slightly among the plants and ranged from 0.79 to 0.83 (Figure 1A). After 48 h of irradiation, the mutants demonstrated a decrease in F_v_/F_m_ from 0.79–0.81 to 0.71–0.74, whereas in the WT plants, this indicator decreased from 0.83 to 0.78. With shorter irradiation periods, the decrease was less pronounced but was noticeable for *pif1pif3pif4pif5* at 32 h (Figure 1, 32 h).

The value of the chlorophyll fluorescence parameter Y(II), which is commonly used as a functional indicator of PSII status, was initially highest in the WT (Figure 1). After irradiation, its values gradually decreased in both the WT and mutants. In the WT, after 16 and 32 h, Y(II) decreased by 1.4 and 1.6 times, respectively. In *pif4* and *pif5*, as well as in WT at 48 h HIL, the Y(II) values decreased by 1.7–2 times compared with the initial values, whereas in *pif1pif3pif4pif5,* the decrease was less significant (1.4 times) (Figure 1).

Exposure to HIL was accompanied by an increase in *NPQ* values. The greatest increase was observed in *pif5* and *pif4pif5* after 16 h and in *pif4* and *pif5* after 48 h. In these cases, NPQ values increased by 1.5 and 2 times, respectively. The greatest decrease in NPQ was observed in the WT after 16 h of irradiation, which was nearly twofold greater than that in the other genotypes (Figure 1C).

**Figure 2:**
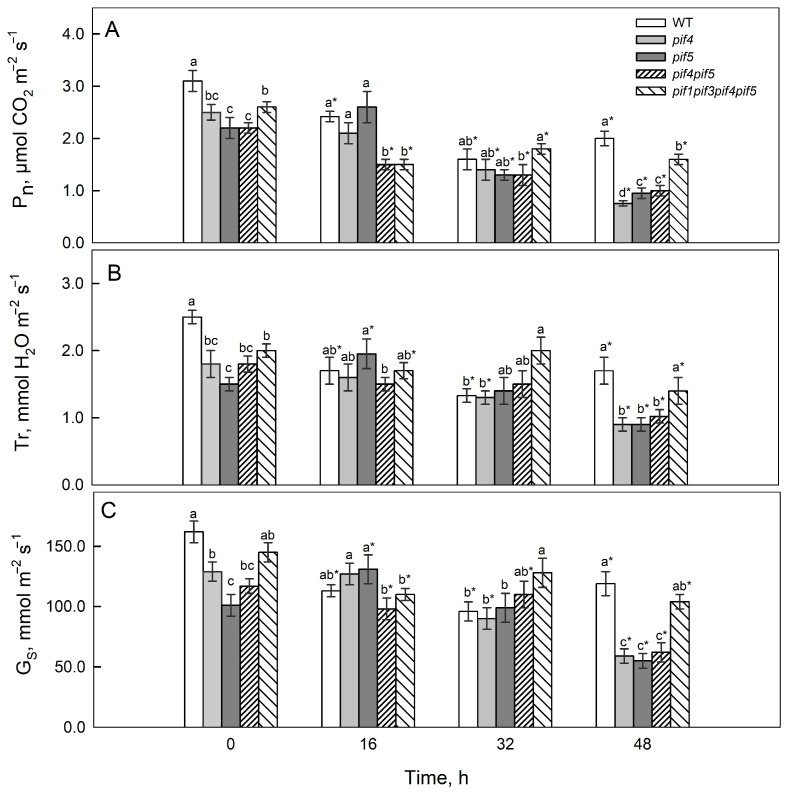
Net photosynthetic (Pn) (**A**) and transpiration (Tr) (**B**) rates and stomatal conductance (g _S_) (**C**) of *A. thaliana* PIF mutants after 16, 32 and 48 h of HIL treatment. Different letters indicate significant differences (*p* ≤ 0.05) according to ANOVA on ranks followed by the Duncan method. Asterisks indicate significant differences within the same genotype at 16, 32, and 48 h (*p* < 0.05; Student’s *t* test). The mean values ± SE are shown, *n* = 7.

Before irradiation, the highest net photosynthesis rate (Pn) was observed in the WT. In *pif4*, *pif5* and *pif4pif5,* the values were close to each other (Figure 2A). After 16 h of HIL, a decrease in the Pn was recorded in the WT by 1.3 times, in *pif4pif5* by 1.5 times, and in *pif1pif3pif4pif5* by 1.7 times. After 32 and 48 h, a decrease in photosynthesis was observed in all the plants. The greatest decrease in Pn (by 3.3 times) and the minimum values were in *pif4* after 48 h; a significant decrease was also noted in *pif5* and *pif4pif5* (by approximately 2.3 times). In WT and *pif1pif3pif4pif5,* the decrease was less pronounced (by 1.5 times).

The stomatal conductance (g_S_) values (Figure 2C) were greater in the WT and the *pif1pif3pif4pif5* mutant than in the other lines. After HIL exposure, a decrease in g_S_ was observed in WT and *pif1pif3pif4pif5* after 16 h_,_ whereas an increase was observed in *pif5.* After 32 h, the g_S_ values decreased in WT and *pif4,* and after 48 h_,_ they decreased in all the lines. Minimum g_S_ values were recorded for all the mutants except *pif1pif3pif4pif5*.

Changes in the transpiration rate (Figure 2B) generally corresponded to the dynamics of stomatal conductance.

### 2.3. Pigment Content

**Figure 3:**
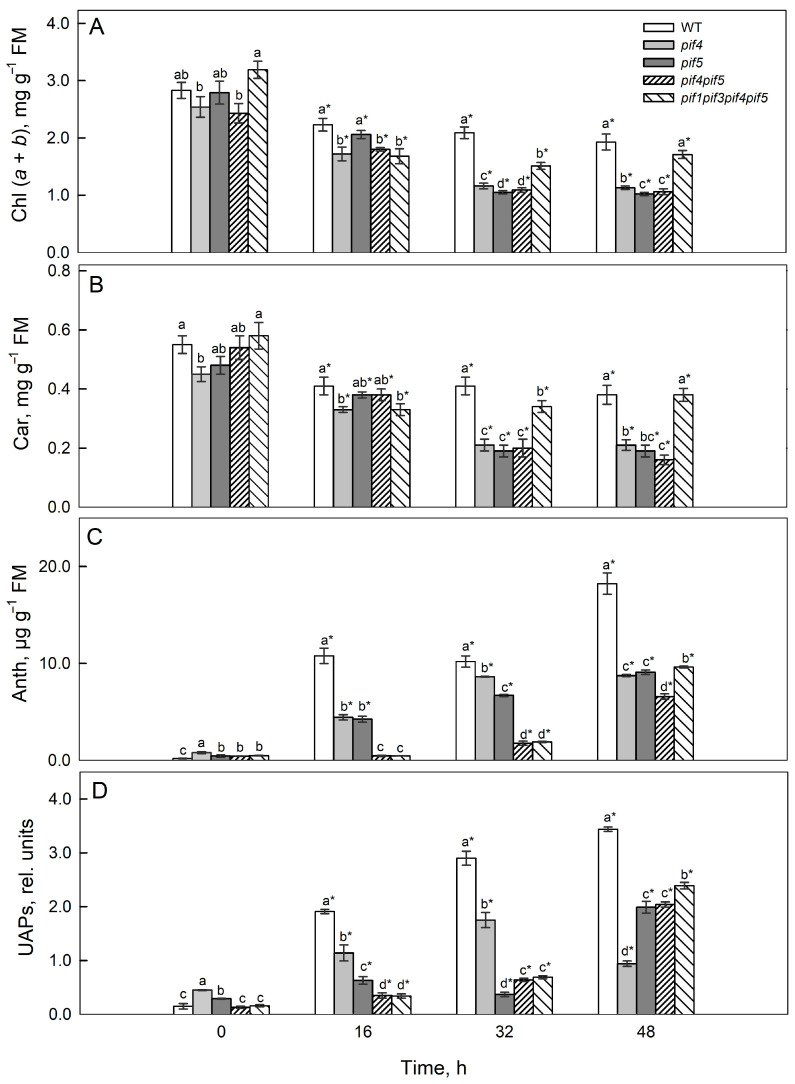
Contents of chlorophyll (*a* + *b*) (Chl) (**A**), carotenoids (Car) (**B**), anthocyanins (Anth) (**C**) and UV-absorbing pigments (UAPs) (**D**) in *A. thaliana* PIF mutants after 16, 32 and 48 h of HIL treatment. Different letters indicate significant differences (*p* ≤ 0.05) according to ANOVA on ranks followed by the Duncan method. Asterisks indicate significant differences within the same genotype at 16, 32, and 48 h (*p* < 0.05; Student’s *t* test). The mean values ± SE are shown, *n* = 3.

Before irradiation, the total chlorophyll content (*a* + *b*) did not differ significantly between the wild type (WT) and the mutant lines. Nevertheless, the quadruple mutant exhibited a tendency toward higher chlorophyll levels relative to the WT, whereas the *pif4* and *pif4pif5* mutants showed slightly lower values, remaining within the range of the WT (Figure 3A). After irradiation, the chlorophyll levels decreased in all the variants. After 16 h, the highest content was retained in the WT and the *pif5* mutant. After 32 and 48 h, the maximum values were observed for WT and *pif1pif3pif4pif5*, whereas the remaining mutants presented relatively low chlorophyll contents.

Initially, the carotenoid content in *pif4* was lower than that in the WT and *pif1pif3pif4pif5* (Figure 3B). After 16 h of irradiation, the carotenoid content decreased in all the variants and was the lowest in *pif4* and *pif1pif3pif4pif5*. After 32 and 48 h, the carotenoid level was greater in the WT and in the *pif1pif3pif4pif5* mutant than in *pif4*, *pif5* and *pif4pif5*. Moreover, after 48 h, the carotenoid content, compared with the initial level, decreased in WT and *pif1pif3pif4pif5* by almost 1.5 times, in *pif4pif5* by 3.5 times, and in *pif4* and *pif5* by 2 and 2.5 times, respectively.

At the initial time point (0 h), no significant differences in the chlorophyll *a/b* ratio were observed among the genotypes. Both the wild-type and *pif* mutant lines presented comparable pigment contents. The (Chl *a + b*)/Car ratio was lower in *pif4pif5, pif5* and *pif1pif3pif4pif5* than in the WT and *pif4* (Table S1).

After 16 h of HIL exposure, distinct differences among the genotypes became evident. The Chl *a/b* ratio decreased in *pif4*, *pif5*, and *pif4pif5* relative to that in the WT. The ratio of the *pif1pif3pif4pif5* mutant was intermediate among those of the WT and other mutants. The (Chl *a* + *b*)/Car ratio reached its highest value in *pif4*, whereas in *pif4pif5* and *pif1pif3pif4pif5,* it was significantly lower (1.33 ± 0.11 and 2.67 ± 0.23, respectively).

After 32 h of irradiation, the WT plants maintained a high Chl *a/b* ratio (2.94 ± 0.15), whereas *pif4*, *pif5*, and *pif4pif5* presented significantly lower values. A similar pattern was observed for the (Chl *a* + *b*)/Car ratio: it was low in *pif1pif3pif4pif5* and high in *pif4* and *pif5*.

After 48 h of HIL exposure, the lowest Chl *a/b* ratios were detected in all the mutants (2.0 ± 0.1) compared with those in the wild type (2.78 ± 0.21). The (Chl *a* + *b*)/Car ratio was the lowest in *pif4pif5* (1.75 ± 0.15), whereas *pif5* maintained relatively high values (6.83 ± 0.72) of the ratio (Table S1). In addition, after 48 h, the Chl *a/b* ratio increased in the WT and decreased in all the mutants except the *pif1pif3pif4pif5* mutant. The reverse pattern for the WT was observed for the (Chl *a* + *b*)/Car ratio.

Before irradiation, the anthocyanin content was highest in *pif4*, exceeding the WT level by 4.4-fold, whereas in the other mutants, it was 1.5–2 times higher (Figure 3C). After 48 h of irradiation, the greatest anthocyanin accumulation was observed in the WT, where the level increased, reaching more than 15 µg g^−1^ FM. In *pif4*, the increase was approximately 8 µg g^−1^ FM, and in *pif5*, *pif4pif5*, and *pif1pif3pif4pif5* mutants, the increase was also significant (approximately 9 µg g^−1^ FM) (Figure 3C).

The UAP content in *pif4* was 3-fold greater than that in WT. In *pif5*, it was almost 2-fold greater, whereas in *pif4pif5* and *pif1pif3pif4pif5*, the values were comparable to those in WT (Figure 3D). After 16 h of irradiation, the UAP level in the WT increased almost 26-fold, whereas in the mutants, it increased only 2–3-fold; the minimum values were observed for *pif4pif5* and *pif1pif3pif4pif5*. After 48 h, the UAP content in *pif5*, *pif4pif5* and *pif1pif3pif4pif5* was 1.5–2-fold lower than that in the WT and 3.5-fold lower than that in *pif4*.

### 2.4. Gene Expression

**Figure 4:**
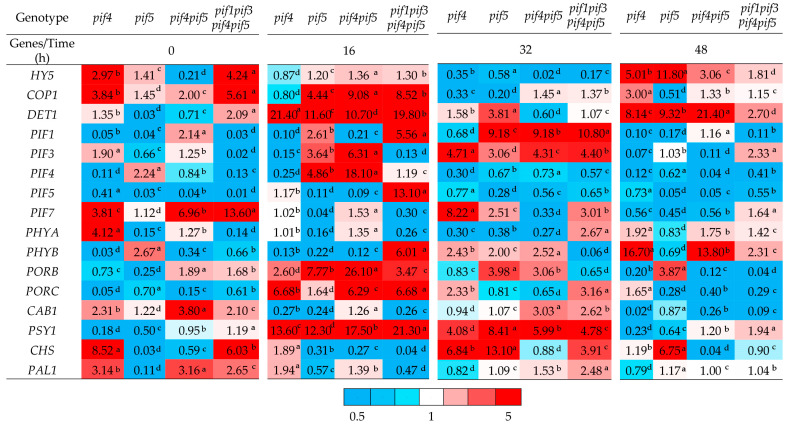
Relative expression levels of light-responsive genes in *A. thaliana pif* mutants under HIL treatment. For each gene, the expression values were normalized to those of *Actin1* as a reference gene. The expression level in the WT at the corresponding time point was taken as 1.0. The data were analyzed via one-way ANOVA. Different letters indicate significant differences according to Duncan’s test (*p* ≤ 0.05). The mean values are shown (*n* = 3). The colour scale represents the relative expression levels of the analyzed genes. White indicates the expression level of the WT, taken as 1.0; blue corresponds to a decrease in expression of at least twofold relative to that of the WT; red indicates a twofold or greater increase in expression compared with that of the WT.

Initially, *PIF* family genes presented distinct alterations across the mutants. *PIF1* expression decreased in *pif4*, *pif5*, and *pif1pif3pif4pif5* but increased in *pif4pif5* (2.14) (Figure 4, 0 h). *PIF3* expression was higher in *pif4* (1.90) and *pif4pif5* (1.25) but strongly suppressed in *pif5* and *pif1pif3pif4pif5* (0.02) (Figure 4, 0 h). *PIF4* expression was reduced in *pif4* (0.11), elevated in *pif5* (2.24), decreased in *pif4pif5* (0.84), and strongly reduced in *pif1pif3pif4pif5* (0.13). *PIF5* expression was suppressed in all the mutants compared with that in the WT. *PIF7* expression was upregulated in the *pif4*, *pif5*, *pif4pif5* (6.96), and *pif1pif3pif4pif5* (13.60) lines.

After 16 h of HIL (Figure 4), *DET1* expression in the mutants markedly increased, increasing more than 11- to 21-fold relative to that in the WT, especially in *pif4* and *pif1pif3pif4pif5. COP1* expression also increased in *pif5* and *pif4pif5*, as well as in *pif1pif3pif4pif5* (4- to 9-fold higher than that in the WT), but not in *pif4* (Figure 4, 16 h). *HY5* levels remained at the WT level in all the mutants.

Among the phytochromes, the expression of the *PHYA* apoprotein gene in *pif4* and the quadruple mutant remained at the wild-type level but decreased in the other lines. Conversely, the expression of the *PHYB* apoprotein gene increased only in the quadruple mutant (by 6-fold) but decreased in the other variants (Figure 4, 48 h).

Pigment metabolism genes were significantly activated after 16 h of irradiation. For example, *PORB* expression was 26-fold greater in *pif4pif5* than in WT. *PORC* expression was also greater in *pif4*, *pif4pif5*, and *pif1pif3pif4pif5* (6–7 times greater than that in WT). The expression of *PSY1* (the gene encoding the key regulatory enzyme of carotenoid biosynthesis) increased in all the mutants (12–21 times greater than that in the WT)*. CAB1* expression decreased in all variants except *pif4*, where it remained at WT levels. The expression of flavonoid biosynthesis pathway genes (*CHS* and *PAL1*) after 16 h was highest in *pif4* (approximately 2-fold greater than that in the WT), whereas in the other lines, it either decreased or remained at the WT level, such as the expression of *PAL1* in *pif4pif5* was 3-fold greater than that in the WT and was moderate in *pif1pif3pif4pif5* (Figure 4).

After 32 h of HIL, *HY5* gene expression decreased in all the lines, especially in *pif4pif5* (Figure 4, 32 h)*. COP1* expression was also decreased in *pif4* and *pif5* but remained at the WT level in *pif4pif5* and *pif1pif3pif4pif5. DET1* expression was high only in *pif5*. Among the *PIF* genes, a general decrease in expression was observed. *PIF1* expression markedly increased in all the mutants (9–11 times higher than that in the WT), except *pif4. PIF3* expression was increased in all the mutants (3–5 times higher than that in the WT). *PIF4* and *PIF5* expression remained low in all the lines. *PIF7* expression was increased in all the mutants except *pif4pif5*; for example, in *pif4,* the expression level was 8-fold greater than that in the WT. *PHYA* expression increased only in *pif1pif3pif4pif5* (2.7-fold greater than that in the WT). *PHYB* expression increased in all the mutants (2–2.5-fold higher than that in the WT), except for *pif1pif3pif4pif5*. Among the pigment metabolism genes, *PORB* expression was significantly increased in *pif5* and *pif4pif5*, and *PORC* expression was increased in *pif4* and *pif1pif3pif4pif5. PSY1* expression increased in all the mutants (4–8-fold higher than that in the WT). *CHS* expression was highest in *pif5* (13-fold higher than that in the WT). The *PAL1* expression level increased only in *pif1pif3pif4pif5* (Figure 4).

After 48 h of HIL (Figure 4, 48 h), *HY5* expression markedly increased in *pif4* and *pif5* (5–12 times higher than that in the WT), but it remained lower in *pif1pif3pif4pif5. DET1* expression was maintained at a high level, especially in *pif4pif5. COP1* expression was high only in *pif4*. The expression of all *PIFs* remained either low or comparable to that of the WT, and the expression of *pif1pif3pif4pif5* was greater than that of the WT (2.3-fold). Among the phytochromes, a pronounced increase in *PHYB* expression was observed in *pif4* and *pif4pif5* (13–17 times higher than that in the WT), whereas *PHYA* expression was elevated in *pif4*. Pigment metabolism genes presented different dynamics. The expression of *PORB* and *PORC* decreased in most of the examined variants, particularly in the *pif4pif5* and quadruple mutants. *PSY1* expression remained elevated only in the quadruple mutant. *CAB1* expression decreased in all the mutants except *pif5*. *CHS* gene expression remained high in *pif5* (6.7-fold higher than that in WT), and *PAL1* expression was close to that in WT (Figure 4).

## 3. Discussion

Phytochromes and some components of phytochrome signalling are involved in the protective mechanisms of the photosynthetic apparatus under the influence of major environmental stressors, particularly HIL [1,27]. However, there is little information on the role and mechanisms of key phytochrome signalling factors, such as PIFs, in these protective processes in response to the development of HIL-induced stress. In this context, the involvement of key PIFs in the adaptive response of *A. thaliana* plants to different HIL exposures was studied. One of the main defence mechanisms under such exposure is nonphotochemical quenching (NPQ), in which part of the absorbed energy is dissipated as heat and the risk of damage to reaction centres is reduced. This is because HIL creates excess excitation in PSII, which increases the formation of reactive oxygen species and reduces the proportion of energy entering photochemistry. These response elements are described in detail in reviews on photoinhibition and redox regulation of metabolism [28,29,30]. Previous studies have demonstrated that the proteins PsbS and zeaxanthin are central elements of the photoprotective machinery associated with nonphotochemical quenching (NPQ) [31,32]. NPQ reflect the combined action of PsbS- and zeaxanthin-dependent mechanisms that safely convert excess absorbed energy into heat, thereby preventing damage to PSII reaction centres.

At the molecular level, *PIF4* and *PIF5* are thought to influence the expression of light-harvesting and pigment-biosynthetic genes such as *CAB1*, *PSY1*, *PORB*, and *PORC*, thereby affecting antenna size and pigment composition, which determine PSII light-absorption capacity. It is assumed that such regulation is possible, although it is likely indirect, and the specific intermediates involved in this mechanism remain to be elucidated. While the exact mechanisms remain to be clarified, the data allow us to hypothesize that *PIF4* and *PIF5* may participate in coordinating photoprotective responses through indirect effects on gene expression, antenna organization, and energy dissipation processes under HIL conditions.

Protective NPQ-related mechanisms include light-induced processes such as energy-dependent quenching, quenching related to photoinhibition, etc., in the *pif4pif 5* mutant after 16 h and 32 h of irradiation and in the *pif4* and *pif5* mutants after 48 h of irradiation.

In our experiments, the response to 16 h of HIL treatment was characterized by a more pronounced decrease in the chlorophyll fluorescence parameter Y(II), as well as in the photosynthetic rate (Pn) and stomatal conductance (gS), in the *pif4 pif5* and *pif1 pif3 pif4 pif5* mutants compared with the WT, whereas NPQ increased (Figure 2 and Figure 3).

These findings suggest that PIF4 and PIF5 influence PSII functional responses under high-intensity light, affecting chlorophyll fluorescence parameters, NPQ dynamics, and stomatal regulation [33]. At later stages of HIL exposure, the *pif4* and *pif5* mutants presented more pronounced decreases in the photosynthetic rate (Pn) and in the chlorophyll-fluorescence parameter Y(II) compared with the WT, which is consistent with the reduced expression of the CAB1 gene and lower levels of photosynthetic pigments in these mutants (Figure 1, Figure 2, Figure 3 and Figure 4). At late stages of HIL exposure, the *pif4*, *pif5* and *pif4pif5* mutants presented a pronounced decrease in the Chl *a/b* ratio compared with that of the wild type, reflecting an increased proportion of chlorophyll *b* relative to chlorophyll *a* and indicating an expansion of the peripheral antenna complexes (Table S1). In contrast, the (Chl *a* + *b*)/Car ratio was markedly reduced in the *pif4*, *pif5* and *pif4pif5* mutants, suggesting either increased carotenoid accumulation or preferential chlorophyll degradation. These pigment composition changes indicate that PIF4 and PIF5 are likely involved in coordinating the regulation of chlorophyll and carotenoid metabolism under high-intensity light. Their absence may disrupt the adjustment of PSII antenna architecture and pigment turnover, leading to a less efficient excitation energy distribution and diminished capacity for thermal energy dissipation, ultimately compromising photoprotection.

Another important mechanism of protection against HIL is the accumulation of low-molecular-weight antioxidants and leaf pigments. These compounds include compounds such as vitamins C and E, glutathione, carotenoids, and different phenolic compounds, including anthocyanins [34]. Leaf pigments can function as cell antioxidants and optical filters that absorb excess light and play important roles in mechanisms of protection from HIL [35].

PIFs play an important role in the regulation of pigment biosynthesis because they regulate the transcription of genes involved in pigment metabolism and the light-harvesting PSII antenna complex (*PSY*, *POR*, *CAB1*) [36]. Under de-etiolation conditions, PIFs can suppress *PSY1* expression [36]; however, despite the induction of the expression of the genes *PSY1*, *PORB* and *PORC* after 16 h of HIL, PIF4 and PIF5 deficiency was accompanied by a more noticeable decrease in chlorophyll and carotenoid content than that in the WT and *pif1pif3pif4pif5* (Figure 2 and Figure 4). Such dynamics may reflect an early adaptive response to reduced light flow absorbed by PSII due to a reduction in antenna size and a decrease in pigment content [37]. In *pif4*, the decrease in *CAB1* transcripts is consistent with a decrease in Y(II), F_v_/F_m_ and Pn values with longer exposure to HIL. The decrease in the content of photosynthetic pigments and probably photosynthetic activity is likely a consequence of a decrease in the size of the antenna and a shift in the balance between the biosynthesis and degradation of pigments and photosynthetic proteins towards their degradation (Figure 1, Figure 2, Figure 3 and Figure 4).

The UAP content was highest after 16 h, which was accompanied by activation of the expression of the *CHS* and *PAL1* genes. Moreover, in all the mutants, the content of these pigments remained lower than that in the WT throughout the experiment, indicating the need for PIF4 and PIF5 for timely activation of antioxidant defence mechanisms and their coordination with photochemical changes under HIL. These observations are consistent with data on the light-dependent regulation of phenolic metabolites, particularly anthocyanins [28]. Since the accumulation of UAPs reflects the synthesis of UV-absorbing phenolic compounds, it can be assumed that increased *CHS* and *PAL1* expression under HIL conditions promotes the formation of such metabolites. In *A. thaliana*, the main noncolored UV-absorbing pigments, along with anthocyanins, are flavonols (such as kaempferol and quercetin) and phenolic acids (including ferulic, p-coumaric, and caffeic acids) [38,39]. These compounds play a key role in protecting photosynthetic tissues from UV-induced oxidative stress. The accumulation of ROS under short-term HIL conditions is associated mainly with increased electron flow through the photosynthetic electron transport chain in chloroplasts. Prolonged HIL leads to stronger suppression of photosynthesis and a reduction in chlorophyll and carotenoid accumulation [28].

PIFs and many other components of light signalling are transcription factors, so changes in their expression interrelatedly affect the activity of numerous target genes that regulate photomorphogenesis, pigment metabolism, and photosynthetic processes. After 16 h of HIL irradiation, the expression of *DET1* and *COP1* increased in the mutants, indicating a weaker light response; simultaneously, the expression of *PSY1*, *PORB*, and *PORC* increased in all the lines, especially in *pif4pif5*; however, the level of photosynthetic pigments decreased (Figure 4), probably due to an increase in the rate of their degradation. In *pif4* and *pif5,* after 48 h, the expression of *HY5* increased, but the expression of *DET1* and COP1 did not decrease; in contrast, their expression, similar to that of a number of *PIFs*, also increased. This expression did not ensure the maintenance of photosynthetic activity and that the pigment content was sufficient for photosynthesis. Moreover, the decrease in *DET1* and *COP1* expression, along with a parallel decrease in *CAB1* in most lines, especially in *pif4*, coincided with a decrease in Y(II), a decrease in F_v_/F_m_ and a decrease in Pn at the late stage of HIL action (48 h) (Figure 2, Figure 3 and Figure 4). These relationships fit into the modern framework of the COP1/DET1–HY5–PIF light signal transduction system [40].

Notably, HIL can also trigger the activation of pathways that increase plant resistance to accompanying environmental factors, such as high temperatures and elevated UV levels, which largely coincide with the pathways of action of HIL; therefore, the induction of the phenylpropanoid pathway and the accumulation of UV-absorbing compounds are considered elements of cross-resistance [41].

## 4. Materials and Methods

### 4.1. Plant Material and Growing Conditions

*Arabidopsis thaliana* seeds of the wild-type (WT, Col-0) and mutant lines (*pif4 CS66043* [42,43,44,45], *pif5 CS66044* [22,42,44,45,46], *pif4pif5 CS68096* [22,42,44], and *pif1pif3pif4pif5 CS66049*) [43,47,48] were obtained from the Arabidopsis Biological Resource Center (ABRC, Columbus, OH, USA). All the lines are homozygous for their respective T-DNA insertions. Their genotypes and phenotypes have been repeatedly verified and reported in multiple independent studies [22,42,43,44,45,49].

Before sowing, the seeds were stratified for three days at 4 °C under humid conditions. The plants were grown in a climate chamber on perlite using Hoagland nutrient solution. The cultivation conditions were as follows: an 8-h photoperiod, day and night temperatures of 23 °C, and a relative air humidity of 75%. The plants were 25 days old at the time of the experiment. To simulate light stress, the plants were exposed to high-intensity light (550 μmol m^−2^ s^−1^) for 0, 16, 24, or 48 h. The 0 h point served as a control for all the analyses. The plants were grown in a controlled-environment growth chamber under cool white LED illumination (Epistar, Xinzhu, Taiwan), which delivers 110 ± 10 µmol photons m^−2^ s^−1^ of photosynthetically active radiation (400–700 nm) at the canopy level. The LED spectrum consisted primarily of blue (~450 nm), green (~550 nm), and red (~630 nm) wavelengths, with no emission in the ultraviolet (<400 nm) or far-red (>700 nm) regions of the spectrum. Light intensity and spectral characteristics were measured using an AvaSpec-ULS4096CL-EVO spectrometer (Avantes, Apeldoorn, The Netherlands). The experiment was conducted using the same light sources at an increased intensity of 550 μmol quanta m^−2^ s^−1^ continuously for 48 h.

### 4.2. Determination of Pigment Content

The chlorophyll (*a* + *b*) and carotenoid contents were determined spectrophotometrically in 80% acetone extracts according to Lichtenthaler 1987 [50]. The absorbance of the samples was measured with a Genesys 10 UV–Vis spectrophotometer (Thermo Fisher Scientific, Waltham, MA, USA) at wavelengths of 470, 646, and 663 nm. The anthocyanins were extracted and determined spectrophotometrically in 1% HCl–methanol extracts according to the methods of Shin et al. 2007 [17].

### 4.3. Photochemical Parameters and Gas Exchange

Chlorophyll fluorescence measurements were performed using a Mini-PAM II fluorometer (Walz, Effeltrich, Germany) on leaves dark-adapted for 20 min. Fluorescence parameters were calculated with WinControl-3 v3.32 software (Walz, Effeltrich, Germany). The following fluorescence levels were recorded: F_0_, Fm, Fv, Fs, F_0_′ and Fm′ [51]. Fm and Fm′ denote the maximum fluorescence in dark-adapted and light-adapted states, respectively, obtained by saturating pulses of 7000 μmol photons m^−2^ s^−1^. Fv represents variable fluorescence (Fv = Fm − F_0_), Fs is the steady-state fluorescence under actinic light, and F_0_ is the initial fluorescence in the dark-adapted state. The chlorophyll fluorescence parameters Fv/Fm and Y(II) = (Fm′ − Fs)/Fm′ were used as functional indicators of PSII status, whereas nonphotochemical quenching (NPQ) was calculated as NPQ = (Fm − Fm′)/Fm [24]. In accordance with recent theoretical developments [25], these parameters are interpreted cautiously and are not considered direct measures of PSII quantum photochemical efficiency.

The net photosynthetic (P_n_) and transpiration (Tr) rates and stomatal conductivity (g_S_) were measured with a CIRAS-2 infrared gas analyser (PP Systems, Amesbury, MA, USA) in a camera area of 2.5 cm^2^ at an illuminance of 120 μmol m^−2^s^−1^ and a CO_2_ concentration of 400 ppm.

### 4.4. RNA Extraction, RT–PCR and Functional Description of Chosen Genes

Total RNA was isolated from leaves using TRIzol (Sigma–Aldrich, St. Louis, MO, USA). The concentration and purity of the samples were assessed spectrophotometrically on a NanoDrop 2000 (Thermo Scientific) [52]. For gene expression analysis, primers for the following genes were used: Elongated Hypocotyl 5, *HY5* NM_001343175.1; Deetiolated 1, *DET1* NM _117086.4; Constitutive Photomorphogenic 1, *COP1* NM _001336418.1; Chalcone synthase, *CHS* NM_121396.4; Phytochrome—Interacting Factor 1, *PIF1* NM_001335667.1; Phytochrome—Interacting Factor 3, *PIF3* NM_100824.3; Phytochrome—Interacting Factor 4, *PIF4* NM_001337007.1; Phytochrome—Interacting Factor 5, *PIF5* NM_001035812.2; Phytochrome—Interacting Factor 7, *PIF7* NM _001345463.1; Phytochrome A, *PHYA* NM _001331843.1; Phytochrome B, *PHYB* NM_001335612.1; Phenylalanine ammonia—lyase 1, *PAL1* NM _129260.3; chlorophyll a/b binding protein 1, *CAB1* NM _102733.3; Phytoene synthase 1, *PSY1* NM_001343483.1; protochlorophyllide oxidoreductase B, *PORB* NM_118879.4; protochlorophyllide oxidoreductase C, *PORC* NM _100243.4; Actin 1, *ACT1* NM_001336664.1 (Table S2). Primer sequences were selected using the NCBI (www.ncbi.nlm.nih.gov), UniProt (www.uniprot.org) and Phytozome (phytozome-next.jgi.doe.gov; accessed 1 February 2025) database by the program VectorNTI Suite 9 (Invitrogen, Waltham, MA, USA). The expression levels were normalized to those of the *Actin1* gene, and the expression value in the WT was taken as 1.

HY5 functions as a central transcriptional activator in light signalling, promoting the expression of genes involved in pigment biosynthesis and photomorphogenic development [53]. DET1 and COP1 act as repressors of light-dependent transcription; their inactivation under high irradiance conditions results in HY5 accumulation and activation of light-responsive pathways [54]. Members of the PIF family mediate the transcriptional output of phytochrome signalling by integrating red and far-red light perception with hormonal and metabolic cues, thus adjusting plant development and stress responses under variable illumination [55]. PHYA and PHYB encode the primary red/far-red photoreceptors responsible for light detection and the regulation of PIF stability. The structural and metabolic genes *CAB1*, *PSY1*, *PORB*, and *PORC* contribute to chlorophyll and carotenoid biosynthesis, determining pigment accumulation and photosynthetic capacity under high-intensity light [56]. CHS and PAL1 participate in the phenylpropanoid pathway, supporting the biosynthesis of flavonoids and other phenolic compounds that mitigate photooxidative stress [57].

### 4.5. Statistical Analysis

Fluorescence and gas exchange measurements were performed on 4–6 plants by one or two developed leaves from each plant. Three plants were used for biochemical and transcriptional analyses, and a pool of developed leaves from each individual plant was frozen in liquid nitrogen. At least three separate measurements were performed for each treatment. Statistical data were analyzed by one-way analysis of variance (ANOVA) followed by the Duncan test for multiple comparisons. Differences were considered significant at *p* < 0.05. Mean values ± SE are presented. In the tables and graphs, statistically significant differences among lines are indicated by different superscript letters.

## 5. Conclusions

Together, PIF4 and PIF5 coordinate the balance among pigment biosynthesis, antenna size, and photosynthetic activity under HIL exposure, linking photochemistry and photosynthesis with stomatal regulation and activation of the phenylpropanoid pathway. These PIFs also ensure that the transcription levels of several studied genes are associated with the physiological changes induced by HIL. Moreover, there was a significant difference in the rate of photosynthesis and the contents of various pigments between the *pif4pif5* and *pif1pif3pif4pif5* mutants. Apparently, both *pif1* and *pif3* mutants are also important for this coordination.

**Figure 5:**
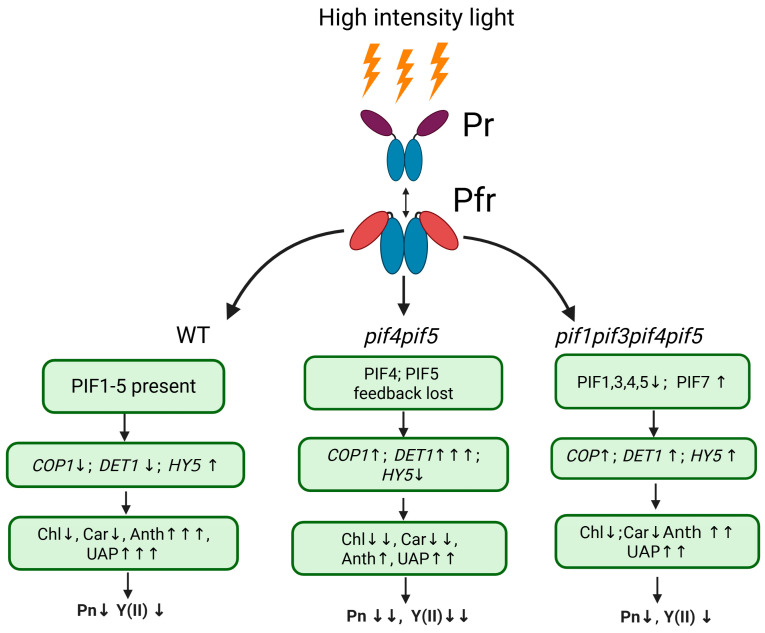
Model summarizing the regulatory functions of PIFs in light signalling and photoprotection under high-intensity light (HIL). Exposure to HIL activates the phytochrome photoreceptor (Pfr), which suppresses PIF activity and thereby contributes to maintaining the balance between light signalling pathways and photoprotective metabolism. In the wild type (WT), the coordinated activity of PIF1–PIF5 supports proper PHYB-mediated feedback regulation, moderate expression of *COP1* and *DET1*, and elevated *HY5* transcription. This regulatory network promotes chlorophyll and carotenoid biosynthesis and favours the accumulation of anthocyanins and UV-absorbing pigments (UAPs). In the *pif4pif5* mutant, disruption of this feedback leads to increased *DET1* and *COP1* expression and reduced *HY5* activity, resulting in lower pigment levels and decreases in photosynthetic performance (Pn) as well as in the chlorophyll fluorescence parameter Y(II), indicating altered PSII functional status. In contrast, the *pif1pif3pif4pif5* mutant shows partial functional compensation through the upregulation of *PIF7* and a moderate recovery of *HY5* expression, accompanied by improved pigment biosynthesis and increased tolerance to HIL. Collectively, these results indicate that PIF4 and PIF5 function as central regulators that connect phytochrome-mediated signalling with pigment metabolism and photosynthetic acclimation under high-light stress. A downward arrow indicates a decrease in the parameter, an upward arrow indicates an increase; the number of arrows reflects the magnitude of the change.

Most likely, to maintain the resistance of the photosynthetic apparatus to HIL and the content of photosynthetic pigments at an earlier stage of light stress development (16 h), deficiencies in PIF4 and PIF5 are not critical, whereas with prolonged exposure to stress, a deficiency of any of these PIFs, especially PIF4, becomes critical. However, a deficiency of PIF1 and PIF3 partially compensates for this criticality. This multidirectional action of these PIFs requires further in-depth study of their functions under stress. Figure 5 provides a schematic summary of the obtained results in the context of previously reported data.

## Data Availability

The data presented in this study are available within the article and its Supplementary Materials. Raw or processed data supporting the findings of this study can be obtained from the corresponding author upon reasonable request.

## Supplementary Materials

The following supporting information can be downloaded at https://www.mdpi.com/article/10.3390/ijms262311660/s1.
