# Supplementary material for: Involvement of Phytochrome-Interacting Factors in High-Irradiance Adaptation
Source: Int J Mol Sci. 2025 Dec 2;26(23):11660. doi: 10.3390/ijms262311660 (PMC12691871; doi:10.3390/ijms262311660)
Supplement: Supplementary file 1 [file ijms-26-11660-s001.zip › S2.pdf]

International Journal of
Molecular Sciences

MDPI

S.2 Table of primers.

Gene Bank ID	Gene description	Gene	Primer 5`-3`	
			Forward	Reverse
NM_001343175.1	bZIP protein Elongated Hypocotyl 5	*HY5*	CAAGCAGCGAGAGGTCATCA	AAAGCATTGACGTTGCAGGTT
NM_117086.4	Deetiolated1	*DET1*	GGAGAGAATGGGTTGCGTCA	GGGGAGGGACTTTGTGACTG
NM_001336418.1	Constitutive Photomorphogenic 1	*COP1*	TCGGGAAGCACTACAAAGGAC	TCTCCACAATCGGACACTGC
NM_121396.4	Chalcone synthase	*CHS*	CCTCTGACACCCACCTTGAC	AGGCAGATAGAAGGCAAGCG
NM_001335667.1	Phytochrome-Interacting Factor 1	*PIF1*	ATCCAACCTCGGGCCAGCCT	TTGGGTCGGGTGGAGACCGC
NM_100824.3	Phytochrome-Interacting Factor 3	*PIF3*	TCCATCTCGAACGGGTTTGG	GACTTGGAACTGTGGTCCGT
NM_001337007.1	Phytochrome-Interacting Factor 4	*PIF4*	ACGGACTCATGGACTTGCTT	CAATGGCTAGGTCCAACGGT
NM_001035812.2	Phytochrome-Interacting Factor 5	*PIF5*	TTGTTGGAGAGGGTTGTTTGGT	ATCGTTCGTGGCTTCTCAGG
NM_001345463.1	Phytochrome-Interacting Factor 7	*PIF7*	GTTTCAGATGTCGTTGCTTGCA	TACCCATAGGAGGGACCATCAT
NM_001331843.1	Phytochrome A	*PHYA*	CCCATTCATCGTCGTCGTCT	GCTCCCAGCAGAAATCAATCG
NM_001335612.1	Phytochrome B	*PHYB*	TCCTTTGAGCGGTATGCGTT	AGACAACAGCGGGAACAATGA
NM_129260.3	Phenylalanine ammonia-lyase 1	*PAL1*	AGGAGGAGTGGACGCTATGT	GAAGTCCGGCGATGTAGGAG
NM_102733.3	Chlorophyll a/b binding protein 1	*CAB1*	CTACCGACCCAGAGGCATTC	CACGAAGCAAAGACTGAAGCAA
NM_001343483.1	Phytoene synthase 1	*PSY1*	CTGCCATGTGGTTCACTGTTT	CCAAATCGCCTTTCGCCTTT
NM_118879.4	Protochlorophyllide Oxidoreductase B	*PORB*	ACGCCTCTCGATGTTTTGGT	CGATTGGTACCGAGAGGTGT
NM_100243.4	Protochlorophyllide Oxidoreductase C	*PORC*	AAGTGATGCGGAGAAGGCAA	GCAGTTCGTTGGTCGTGTCT
NM_001336664.1	Actin 1	*ACT1*	CGAACACACACCCCAATTACG	CATCAGCCATTTTCTTCTACGCA
