# Supplementary material for: Involvement of Phytochrome-Interacting Factors in High-Irradiance Adaptation
Source: Int J Mol Sci. 2025 Dec 2;26(23):11660. doi: 10.3390/ijms262311660 (PMC12691871; doi:10.3390/ijms262311660)
Supplement: Supplementary file 1 [file ijms-26-11660-s001.zip › Table S1.pdf]

Table S1. Changes in chlorophyll and carotenoid ratios in *Arabidopsis thaliana* wild-type and *pif* mutant lines under high-intensity light (HIL) exposure.

	WT	*pif4*	*pif5*	*pif4,5*	*pif1pif3pif4pif5*
	**0**				
Chl a/b	2.22±0.12a	2.48±0.18a	2.32±0.16a	2.42±0.13a	1.95±0.14a
Chl (a+b)/Car	8.09 ± 0.58a	7.94± 0.69a	5.81± 0.45b	4.52± 0.25b	4.83± 0.33b
	**16**				
Chl a/b	2.91±0.16a	2.07±0.17b	1.90±0.06b	2.01±0.10b	2.29±0.27ab
Chl (a+b)/Car	5.44± 0.42b	7.17± 0.44a	3.38± 0.37c	1.33± 0.11d	2.67± 0.23c
	**32**				
Chl a/Chl b	2.94±0.15a	1.97±0.12b	1.91±0.06b	1.96±0.16b	2.60±0.17ab
Chl (a+b)/Car	4.64±0.36b	7.17± 0.85a	8.45± 0.86a	6.64±0.73ab	2.80± 0.17c
	**48**				
Chl a/Chl b	2.78± 0.21a	2.00± 0.10b	1.93± 0.07b	1.87± 0.12b	2.08± 0.09b
Chl (a+b)/Car	4.02± 0.41b	5.72± 0.71ab	6.83± 0.82a	1.75± 0.15c	4.50± 0.36b
